# Iraq Mass Gathering Preparedness and Public Health Recommendations

**DOI:** 10.2196/15886

**Published:** 2020-04-08

**Authors:** Mohannad Al Nsour

**Affiliations:** 1 Global Health Development/Eastern Mediterranean Public Health Network Amman Jordan

**Keywords:** mass gathering, Iraq

Iraq is the host to the largest Eastern Mediterranean Region’s religious mass gathering. In the last decade, the number of people visiting Karbala on the anniversary of Imam Husseyn’s death has increased considerably from year to year. According to 2014 estimates, Karbala City has a local population of approximately 1.1 million individuals in an area of approximately 43.7 km^2^ [1]. Millions of people gather at the “Arbaeenia” gathering in Karbala to mark this important event. The approximate number of visitors has increased from 3 million individuals in 2003 to 25 million in 2016, with about 20% coming from countries external to Iraq [2].

As of the 2014 anniversary, preventive measures such as the request for visit permit and proof of vaccination upon entry to Iraq were not in place. However, many sectors are involved in the gathering’s proceedings once the city starts welcoming its visitors. The Operations Department at the Iraq Ministry of Health (MOH) and the Health Directorates in Karbala, Najaf, Babel, Aldwanya, Thi Qar, Wassit, and Baghdad (ie, Karkh and Rusafah) contribute to the local planning before the event. Medical services are provided by primary health care centers from the MOH and governmental and nongovernmental health clinics. The local municipalities provide water and hygiene services, and the Sacred Al Abbas Mosque and the Sacred Al Husayn Mosque nongovernmental authorities provide accommodations, covers, food, and medical services.

In the face of the high volume of population movement, the changing date of the anniversary, and short latency, public health authorities need to have preparedness plans and resources to effectively manage the additional pressure on the country’s system. Although the Iraq Ministry of Health has been passing the test of safely caring for the large number of visitors every year, it is presented with challenges of providing quality health services and mitigating the increasing risks.

In reviewing the literature of Iraqi mass gatherings, it becomes apparent that the scale of the health strain is not quantified, and the gaps are not identified. In view of the challenges presented by this mass gathering, whether they are related to quantity or quality of services provided to attendees, public authorities and supporting organizations should be ready to accommodate masses throughout the event including pre-event preparation and postevent activities.

Keeping abreast of the economic and political situation in Iraq, the Eastern Mediterranean Public Health Network (EMPHNET) with Iraq Ministry of Health and support from the US Department of State’s Biosecurity Engagement Program and Centers for Disease Control and Prevention launched a mass gathering project for the Field Epidemiology Training Program and public health professionals working at the Iraq Ministry of Health from different public health departments. The major aim of this mass gathering project was to strengthen the public health system efforts in accommodating masses and reducing morbidity and mortality during the anniversary of Imam Husseyn’s death. The project encompassed three phases and resulted in eight manuscripts. The first phase was conducting an introductory workshop to public health in mass gatherings for field epidemiologists and other health professionals. The second phase focused on the implementation of operational research and holding a policy brief meeting on the findings of the research. The third phase entailed conducting a scientific writing workshop in preparation for manuscripts on the research carried out around the 2014 anniversary of Imam Husseyn’s death.

This e-collection [3-10] of the EMPHNET Iraq Mass Gathering Project (2014-2015) was published to promote better readiness and identify any health risk management gaps. Additionally, these publications will help proliferate the much-needed research and literature on public health issues related to mass gathering in the Middle East. The publications included were peer reviewed by Baghdad University, EMPHNET, and other external technical experts. The articles presented in this supplement will hopefully provide data to initiate better preparedness and planning for future mass gatherings in Iraq.

## Abbreviations

EMPHNET: Eastern Mediterranean Public Health Network
MOH: Ministry of Health
